# Peripheral blood monocytes could be associated with brain metastasis and affect patient prognosis in breast cancer: a retrospective study

**DOI:** 10.3389/fonc.2026.1807911

**Published:** 2026-05-21

**Authors:** Jihwan Yoo, Hun Ho Park, Seo Yeon Kim, Sung Jun Ahn, Jungho Ahn, Jiwoong Oh, Jaejoon Lim

**Affiliations:** 1 Department of Neurosurgery, Brain Tumor Center, Gangnam Severance Hospital, Seoul, Republic of Korea; 2 Department of Radiology, Gangnam Severance Hospital, Seoul, Republic of Korea; 3 Department of Neurosurgery, Severance Hospital, Yonsei University, College of Medicine, Seoul, Republic of Korea; 4 Department of Biophysics, Institute of Quantum Biophysics, Sungkyunkwan University, Suwon, Republic of Korea; 5 Department of Neurosurgery, Bundang CHA Medical Center, CHA University College of Medicine, Seongnam, Republic of Korea

**Keywords:** brain metastasis, breast cancer, monocyte, monocyte-to-lymphocyte ratio, survival

## Abstract

**Objective:**

The aim of this study was to test the hypothesis that changes in peripheral blood monocyte-related ratios, particularly the lymphocyte-to-monocyte ratio (LMR), at the time of brain metastases (BM) diagnosis are associated with survival outcomes in patients with breast cancer.

**Methods:**

This study included 117 patients with breast cancer who developed BM from 2008 to 2023. We analyzed peripheral blood counts at primary diagnosis and at BM diagnosis to examine changes in monocyte count. The impact on survival was assessed using the monocyte-related ratios.

**Results:**

The absolute monocyte count (AMC) increased 2.11-fold at BM diagnosis compared with initial breast cancer diagnosis (p = 0.038). The high LMR group had a median survival of 20.0 months (95% CI 15.2–28.5) compared with 5.13 months (95% CI 3.9–7.5) in the low group (log-rank p < 0.001). Similarly, the high platelet-to-monocyte ratio (PMR) group had a median survival of 14.17 months (95% CI 7.0–18.7) versus 5.73 months (95% CI 3.6–9.9) in the low group (log-rank p = 0.028). Prognostic factors significantly associated with poorer survival included triple-negative breast cancer subtype, low LMR, and number of BMs, while age and extracranial metastasis exerted minimal impact.

**Conclusion:**

Changes in AMC and monocyte-related ratios at the time of BM diagnosis are associated with the development of BM and can impact survival outcomes in patients with breast cancer. These findings suggest that monocyte dynamics may serve as exploratory prognostic markers, which require validation in prospective studies.

## 1 Introduction

Brain metastases (BM) represent one of the most devastating complications of breast cancer, occurring in up to 30% of patients during the disease course and contributing to significant morbidity and mortality (1, 2). Advances in systemic therapy have improved survival, but the incidence of BM continues to rise (3).

While tumor-intrinsic factors and microenvironmental changes have been implicated in BM development, the contribution of circulating immune cells remains less well defined. *In vivo* studies have demonstrated that circulating tumor cells (CTCs) can lodge within the cerebral vasculature and occasionally mimic vascular occlusion, while a subset undergoes extravasation into brain parenchyma (approximately one-third in experimental models (4). However, the role of host immune cells in this process is incompletely understood.

Recent experimental studies have highlighted the multifaceted roles of monocytes in the metastatic process (5). Monocytes, particularly the inflammatory subset, have been shown to facilitate the extravasation of CTCs across the blood-brain barrier (BBB) by secreting factors like CCL2, which increases vascular permeability (6, 7). Furthermore, monocyte-derived macrophages are known to contribute to the formation of a pre-metastatic niche, providing a supportive environment for tumor seeding and growth in the brain parenchyma (8). Building on these biological findings, the clinical significance of systemic immune cells has gained increasing attention. Alterations in monocyte counts and lymphocyte-to-monocyte ratio (LMR) have been associated with outcomes in several malignancies, yet their role in BM from breast cancer has not been systematically examined (9–11).

Given these considerations, we hypothesized that dynamic changes in monocytes and monocyte-related ratios from primary diagnosis to BM onset may reflect clinically relevant biology and impact survival outcomes in breast cancer patients with BM.

## 2 Materials and methods

### 2.1 Patients and laboratory measurements

Among patients diagnosed with primary BRCA, we included 211 with BM confirmed by magnetic resonance imaging from 2008 to 2023. From the study cohort, we excluded 52 individuals who had concurrent diagnoses of both BRCA and BM, 3 with sarcoma, 18 who had been diagnosed with primary BRCA in the distant past (lacking complete blood count [CBC] data), and 21 who had undergone surgery for primary BRCA at another hospital and therefore did not have initial CBC information available. Finally, 117 patients were included, and age, Karnofsky performance score (KPS), extracranial metastasis (ECM), number of BM, subtype, and treatment history were analyzed. Survival was evaluated as overall survival, defined as the time from BM diagnosis to death. Peripheral blood counts were obtained from electronic medical records. All analyses were performed at the certified clinical laboratory of Gangnam Severance Hospital, minimizing inter-laboratory variation.

### 2.2 Monocyte-related values

Absolute counts including the AMC, absolute neutrophil count (ANC), absolute lymphocyte count (ALC), and absolute platelet count (APC) were recorded. We used the initial CBC profile obtained at the time of breast cancer diagnosis, prior to any surgical or other therapeutic interventions, to ensure baseline values. Furthermore, relative ratios with monocytes, including the neutrophil-to-monocyte ratio (NMR), LMR, and platelet-to-monocyte ratio (PMR) were calculated.

For all patients, the degree of elevation in LMR at BM diagnosis (BM LMR/primary BRCA LMR) was calculated relative to LMR at primary BRCA diagnosis, and patients were categorized into the high LMR elevated group and low LMR elevated group according to the optimal cutoff value calculated using *maxstat* R packages. These two groups were statistically compared with respect to age, ECM, KPS, number of BMs, and subtypes, each of which could influence prognosis, as well as craniotomy, radiotherapy, chemotherapy, targeted therapy, and immunotherapy. Survival analysis according to the degree of PMR elevation was also performed using the same method.

### 2.3 Statistical analysis

Comparisons between AMC, ANC, ALC, APC, NMR, LMR, and PMR at the time of primary BRCA diagnosis and BM diagnosis were calculated using a paired Wilcoxon signed-rank test. Continuous variables between the high and low LMR elevated groups were analyzed using Student’s t-test, whereas categorical variables were analyzed using chi-square or Fisher’s exact test. Treatment variables, including craniotomy, radiotherapy, chemotherapy, targeted therapy, and immunotherapy, were included as covariates in the survival analysis. Each factor was first tested in univariate Cox regression, and variables showing statistical or clinical relevance were subsequently evaluated in the multivariate Cox regression model. For overall survival, Kaplan–Meier survival analysis was performed using the *survival* R-package, and log-rank scores were calculated for comparison between the two groups. Cox regression analysis was performed using the *survival* R-package, focusing on variables known to influence prognosis: age, KPS, ECM, subtype, number of BMs, and the high LMR elevated group. All statistical analyses were performed using the R software version 4.1.3 (R Foundation for Statistical Computing, Vienna, Austria). Statistical significance was set at P <0.05.

## 3 Results

### 3.1 Patient demographics

The patient selection process is summarized in a STROBE-style flowchart (**Supplementary Figure 1**), which depicts the inclusion and exclusion criteria applied to arrive at the final study cohort of 117 patients. **Table 1** summarizes the demographics of the 117 patients included in the study. The average age at diagnosis of BM was 51.7 years, and all patients were female. The subtypes were distributed as follows: 38 patients (32.5%) had Luminal-like, 30 patients (25.6%) were HER2-positive, and 49 patients (41.9%) had triple-negative breast cancer (TNBC). ECM presence was detected in 81 patients (69.8%). The most frequently observed KPS was between 70 and 80, which was exhibited by 74 patients (63.2%). The average number of BMs observed was 8.1 per patient. Chemotherapy was administered to 115 patients (98.3%), while 43 patients (37.4%) received HER2-targeted therapy. Craniotomy for BM and radiotherapy were performed in 46 (39.7%) and 94 (81.0%) patients, respectively.

**Table 1:** Patient demographics.

Variables	Total (n = 117)
Age at diagnosis of primary BRCA (yrs), mean ± SD	48.7 ± 9.3
Age at diagnosis of BM (yrs), mean ± SD	51.7 ± 9.4
Time interval between BRCA and BM (months), mean ± SD	36.2 ± 29.2
Sex	
Female, n (%)	117 (100.0%)
Subtype	
Luminal-like, n (%)	38 (32.5%)
HER2+, n (%)	30 (25.6%)
TNBC, n (%)	49 (41.9%)
Extracranial metastasis, n (%)	81 (69.8%)
KPS, n (%)	
< 70	6 (5.1%)
70–80	74 (63.2%)
90–100	37 (31.6%)
Number of BM, n (%)	8.1 ± 13.3
multiple metastases (n >3)	62 (53.0%)
oligo metastases (n = 2, 3)	21 (17.9%)
single metastasis (n = 1)	34 (29.1%)
Distant metastasis before BM	59 (50.4%)
Metastasis to Lung	40 (34.2%)
Metastasis to Liver	12 (10.3%)
Metastasis to Liver	12 (10.3%)
Chemotherapy, n (%)	115 (98.3%)
Hormone therapy, n (%)	33 (28.2%)
Targeted therapy, n (%)	43 (37.4%)
Immunotherapy, n (%)	8 (6.8%)
Craniotomy, n (%)	46 (39.7%)
Radiotherapy, n (%)	94 (81.0%)

BRCA, breast cancer; BM, brain metastases; HR, hormone receptor; HER2, human epidermal growth factor receptor 2; KPS, Karnofsky performance status; SD, standard deviation; TNBC, triple-negative breast cancer.

### 3.2 Blood cell counts (monocyte, lymphocyte, neutrophil, platelet) at primary BRCA and BM diagnoses

**Figure 1:**
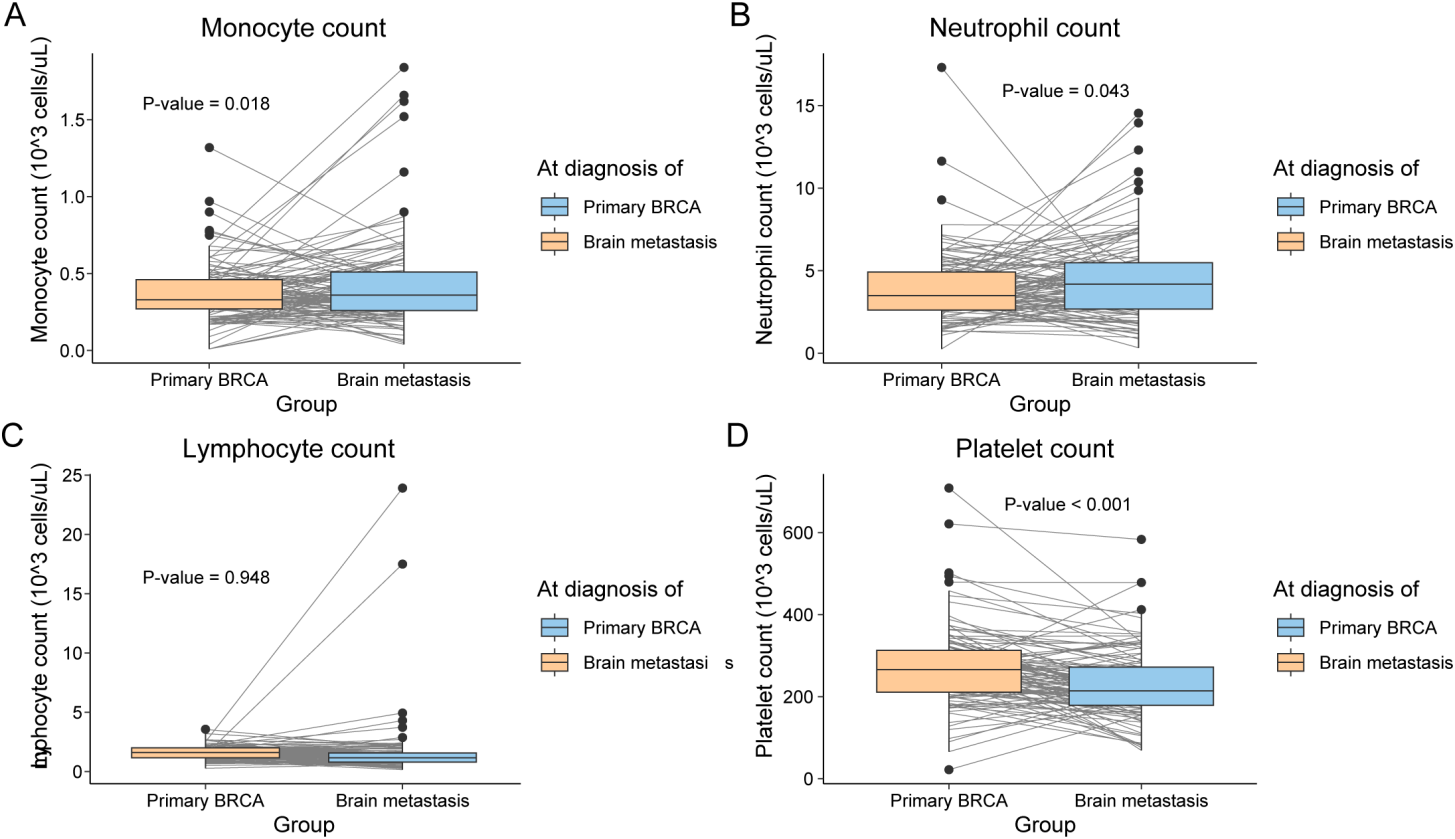
Absolute blood cell counts at diagnosis of primary BRCA and BM. **(A)** Absolute monocyte count (10^3^ cells/µL). **(B)** Absolute neutrophil count (10^3^ cells/µL). **(C)** Absolute lymphocyte count (10^3^ cells/µL). **(D)** Absolute platelet count (10^3^/µL). BRCA, breast cancer; BM, brain metastasis.

The distribution of blood cell counts in peripheral blood is summarized and presented in **Figure 1**. The AMC (cells/μL) was significantly elevated at the time of BM diagnosis when compared with values at primary BRCA diagnosis (primary BRCA: 366.0 ± 192.1; BM: 435.1 ± 302.6; p = 0.038). Conversely, the APC (cells/μL) was significantly reduced at BM diagnosis when compared with that at primary BRCA (primary BRCA: 272.0 ± 98.1; BM: 228.7 ± 87.0; p <0.001). The ANC (cells/μL) (primary BRCA: 3940.9 ± 2154.6; BM: 4541.9 ± 2603.3; p = 0.056) and ALC (cells/nL) (primary BRCA: 1598.4 ± 576.0; BM: 1614.4 ± 2674.1; p = 0.950) did not show any significant changes.

### 3.3 Monocyte-related values at primary BRCA and BM diagnosis

**Figure 2:**
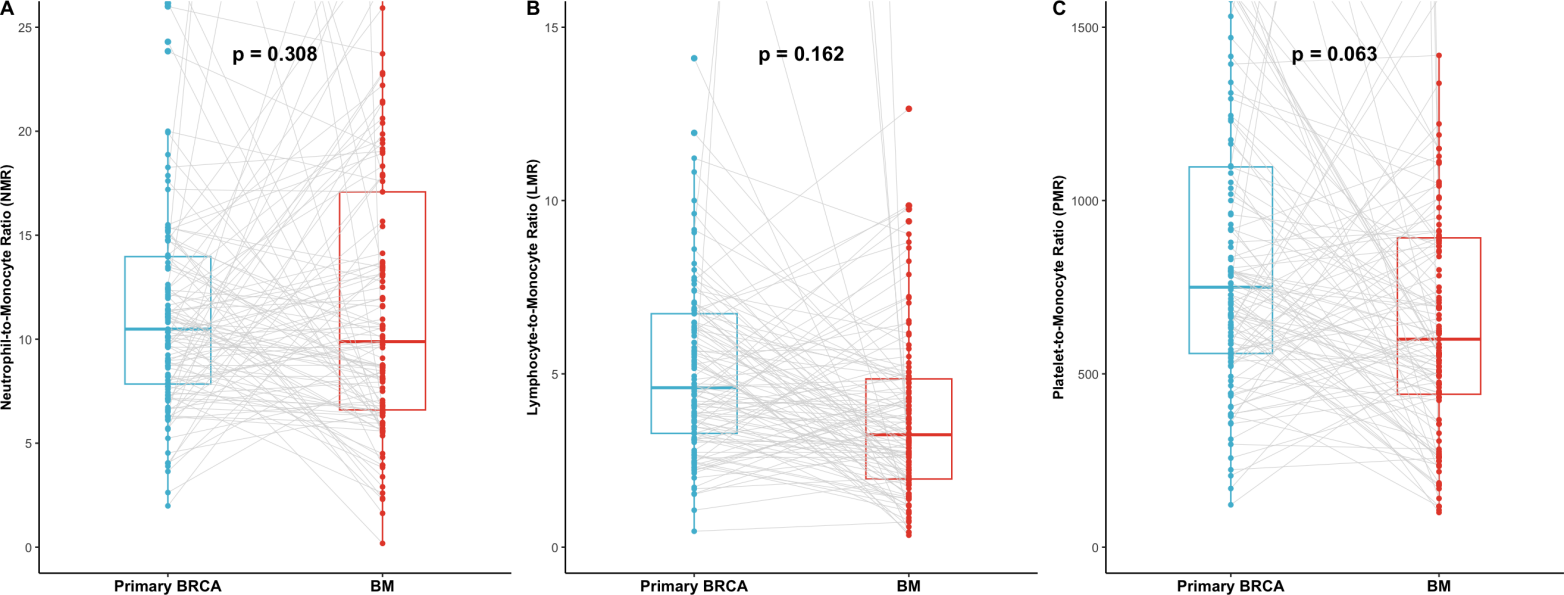
Monocyte-to-related blood cells ratio at diagnosis of primary BRCA and BM. **(A)** Neutrophil-to-monocyte ratio (NMR) (%). **(B)** Lymphocyte-to-monocyte ratio (LMR) (%). **(C)** Platelet-to-monocyte ratio (PMR) (%). BRCA, breast cancer; BM, brain metastasis.

The longitudinal analysis of monocyte-related ratios revealed a downward trend at the time of BM diagnosis compared to the primary BRCA diagnosis (**Figure 2**). The LMR decreased from 7.69 ± 17.31 at primary BRCA diagnosis to 5.08 ± 10.86 at the time of BM (p = 0.162). Similarly, the PMR showed a decrease from 1757.29 ± 5603.90 in primary BRCA to 772.42 ± 736.54 in BM (p = 0.063). The NMR also changed from 19.18 ± 52.79 to 13.96 ± 15.84 (p = 0.308). Although these overall longitudinal changes did not reach statistical significance due to the high variability of the hematologic parameters, the dynamic shift toward a lower LMR was associated with distinct prognostic outcomes, as further analyzed in Section 3.5.

### 3.4 Survival analysis by LMR and PMR elevation and Cox regression analysis

**Figure 3:**
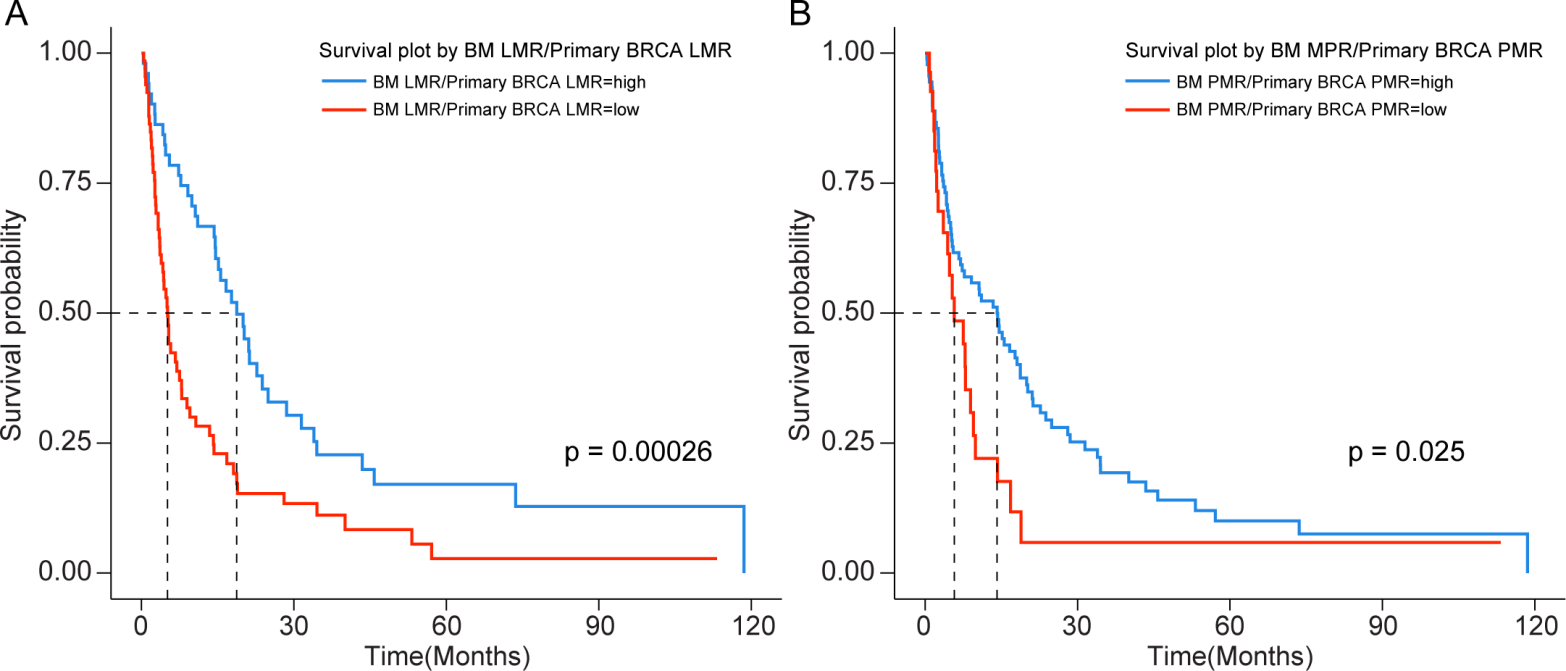
Kaplan–Meier survival analysis based on monocyte-related ratio changes from primary BRCA to BM diagnosis. **(A)** Survival according to BM LMR/primary BRCA LMR groups: median OS 20.0 months (95% CI 15.2–28.5) in the high group vs. 5.13 months (95% CI 3.9–7.5) in the low group (log-rank p < 0.001); HR 0.43 (95% CI 0.28–0.65), p < 0.001. **(B)** Survival according to BM PMR/primary BRCA PMR groups: median OS 14.17 months (95% CI 7.0–18.7) in the high group vs. 5.73 months (95% CI 3.6–9.9) in the low group (log-rank p = 0.028); HR 0.58 (95% CI 0.35–0.94), p = 0.028.

Of the 117 patients included in the study, 96 succumbed to the disease, with a median survival of 9.5 months. The optimal cutoff value for the ratio of BM LMR to primary BRCA LMR was determined as 1.322999. Based on this threshold, 66 patients were classified into the BM LMR/primary BRCA LMR high group, while 51 patients were assigned to the BM LMR/primary BRCA LMR low group. The high LMR group (n=51) had a median survival of 20.0 months (95% CI 15.2–28.5) compared with 5.13 months (95% CI 3.9–7.5) in the low group (n=66; log-rank p < 0.001). In univariate Cox regression, high LMR was associated with better survival (HR 0.43, 95% CI 0.28–0.65, p < 0.001; **Figure 3A**).

The optimal cutoff value for the ratio of BM PMR to primary PMR was 2.354207. Using this criterion, 27 patients were categorized into the BM PMR/primary BRCA PMR high group, and 90 patients were assigned to the BM PMR/primary BRCA PMR low group. Similarly, the high PMR group (n=90) had a median survival of 14.17 months (95% CI 7.0–18.7) compared with 5.73 months (95% CI 3.6–9.9) in the low group (n=27; log-rank p = 0.028). In univariate Cox regression, high PMR was associated with improved survival (HR 0.58, 95% CI 0.35–0.94, p = 0.028; **Figure 3B**).

Cox regression analysis was conducted to evaluate the impact of known prognostic factors, including age, KPS, number of BMs, ECM, subtype, and LMR and PMR groups, on prognosis (**Tables 2**, **3**). In the univariate Cox regression analysis, the subtype (TNBC), number of BMs, LMR group, and PMR group showed statistical significance, whereas age, KPS, and ECM did not exhibit statistical significance. However, in the multivariate Cox regression analysis, only the subtype (TNBC), number of BMs, and LMR were statistically significant.

**Table 2:** Univariate and multivariate Cox regression analyses of clinical variables for survival of patients.

Variables	Univariate regression			Multivariate regression		
	HR	95% CI	p-value	HR	95% CI	P-value
Age at BM, yrs	0.999	0.975–1.024	0.9495			
Subtype						
Luminal-like	ref			ref		
HER2+	0.726	0.418–1.261	0.256	0.811	0.461–1.426	0.467
TNBC	1.713	1.075–2.729	0.024	2.092	1.292–3.389	0.003^**^
KPS						
90–100	ref					
70–80	1.484	0.948–2.322	0.084			
<70	0.885	0.309–2.535	0.820			
BM number						
Single (n=1)	ref			ref		
Oligo (n=2,3)	2.570	1.339–4.933	0.005^**^	2.035	1.044–3.966	0.037
Multiple (n>3)	2.915	1.720–4.940	<0.001^***^	2.730	1.593–4.679	0.0003^**^
ECM	1.579	0.988–2.522	0.056			
High BM PMR elevation	0.596	0.362-0.979	0.041*	0.811	0.462-1.424	0.466
High BM LMR elevation	0.434	0.285-0.662	<0.001^***^	0.418	0.256-0.684	<0.001^***^

BM, brain metastases; CI, confidence interval; ECM, extracranial metastasis; HER2+, human epidermal growth factor receptor 2-positive; HR, hazard ratio; KPS, Karnofsky performance status; PMR, platelet-to-monocyte ratio; LMR, lymphocyte-to-monocyte ratio; SD, standard deviation; TNBC, triple-negative breast cancer.

* : p < 0.05; ** : p < 0.01; *** : p < 0.001.

**Table 3:** Multivariate Cox regression including clinical and treatment covariates.

Variables	HR	95% CI (Lower)	95% CI (Upper)	P-value
Age	1.0076	0.9804	1.0356	0.588695
KPS				
90-100	ref			
70-80	1.1325	0.6855	1.8712	0.627112
<70	0.994	0.2994	3.2998	0.992203
BM number				
single (n=1)	ref			
oligo (n=2,3)	2.4759	1.1763	5.2114	0.016958
multiple (n>3)	2.4497	1.3005	4.6145	0.00555^***^
Subypte				
HER2	ref			
luminal-a	1.0533	0.3461	3.2059	0.927164
luminal-b	0.8892	0.3733	2.1179	0.790812
TNBC	2.0635	0.7974	5.3399	0.135359
ECM	0.6383	0.3501	1.1639	0.143002
Conventional chemotherapy	7.5915	0.7187	80.1862	0.091922
Targeted therapy	0.6676	0.2701	1.6501	0.381451
Hormone therapy	1.2174	0.4527	3.274	0.696734
immunotherapy	0.8719	0.3214	2.3655	0.78781
craniotomy	0.6283	0.3396	1.1627	0.138917
High BM LMR elevation	0.4084	0.2398	0.6955	0.000978^***^
High BM PMR elevation	0.8105	0.4313	1.523	0.513918

BM, brain metastases; HR, Hazard ratio; CI, confidence interval; ECM, extracranial metastasis; HER2+, human epidermal growth factor receptor 2-positive; HR, hazard ratio; KPS, Karnofsky performance status; PMR, platelet-to-monocyte ratio; LMR, lymphocyte-to-monocyte ratio; SD, standard deviation; TNBC, triple-negative breast cancer.

*** : p < 0.001.

### 3.5 Comparison of clinical variables between BM LMR/primary BRCA LMR high and low group

Considering clinical variables such as age, subtype, and KPS, no statistically significant differences were observed between the BM LMR/primary BRCA LMR high group and the BM LMR/primary BRCA LMR low group (**Table 4**). In addition, we examined potential confounding factors that could influence peripheral blood counts. As shown in **Table 4**, the timing of blood draws relative to primary breast cancer and BM diagnosis, recent systemic treatments, and the use of antibiotics or corticosteroids did not significantly differ between the two groups. However, ECM was significantly more prevalent in the BM LMR/primary BRCA LMR high group, detected in 80% of these patients (p = 0.013). There was no statistically significant difference in the timing of therapeutic agent administration, which can impact the blood cell count, between the two groups. In addition, analysis of blood cell components showed that the LMR-low group was characterized by a marked increase in monocytes with relative lymphocyte reduction, whereas the LMR-high group exhibited minimal monocyte change with relative preservation of lymphocytes (**Table 5**).

**Table 4:** Comparison of clinical variables between BM LMR/Primary BRCA LMR high group and BM LMR/Primary BRCA LMR low group.

Variables	BM LMR/primary BRCA LMR low group, (N=51)	BM LMR/primary BRCA LMR High group, (N=66)	P value
Age at diagnosis of BM (yrs), mean ± SD	50.6 ± 8.7	52.5 ± 9.8	0.288
Time interval between primary BRCA and BM (yrs), mean ± SD	36.6 ± 31.2	35.9 ± 27.9	0.891
Subtype			0.091
Luminal-like, n (%)	13 (25.5%)	25 (37.9%)	
HER2+, n (%)	18 (35.3%)	12 (18.2%)	
TNBC, n (%)	20 (39.2%)	29 (43.9%)	
Hormone receptor, n (%)	13 (25.5%)	25 (37.9%)	0.223
HER2, n(%)	25 (49.0%)	27 (40.9%)	0.492
Extracranial metastasis, n (%)	29 (56.9%)	52 (80.0%)	0.013^*^
KPS, n (%)			0.158
<70	1 (2.0%)	5 (7.6%)	
70–80	30 (58.8%)	44 (66.7%)	
90–100	20 (39.2%)	17 (25.8%)	
Number of BM, n (%)			0.337
Single metastasis (n=1)	18 (35.3%)	16 (24.2%)	
Oligo metastases (n=2, 3)	7 (13.7%)	14 (21.2%)	
Multiple metastases (n>3)	26 (51.0%)	36 (54.5%)	
Chemotherapy before BM, n (%)	49 (96.1%)	66 (100.0%)	0.366
Hormone therapy before BM, n (%)	13 (25.5%)	20 (30.3%)	0.714
Targeted therapy before BM, n (%)	20 (40.0%)	23 (35.4%)	0.754
Immunotherapy before BM, n (%)	1 (2.0%)	6 (9.1%)	0.223
Craniotomy, n (%)	22 (43.1%)	24 (36.9%)	0.626
Radiotherapy, n (%)	44 (86.3%)	50 (76.9%)	0.300
Time interval from blood draw on primary BRCA diagnosis (days), mean ± SD	0.9 ± 3.0	1.9 ± 4.0	0.131
Time interval from blood draw on BM diagnosis (days), mean ± SD	3.3 ± 9.1	5.6 ± 10.1	0.191
Time interval from last chemotherapy before BM (months), mean ± SD	11.5 ± 11.6	8.3 ± 15.1	0.219
Time interval from last hormone therapy before BM (months), mean ± SD	10.8 ± 17.4	14.7 ± 11.9	0.450
Time interval from last targeted therapy before BM (months), mean ± SD	8.6 ± 11.8	7.7 ± 10.8	0.794
Time interval from last immunotherapy before BM (months), mean ± SD	0.3	2.6 ± 2.6	NA
Antibiotics use, n (%)	6 (9.1%)	2 (3.9%)	0.466
Steroid use, n (%)	6 (9.1%)	1 (2.0%)	0.223

BM, brain metastasis; BRCA, breast cancer; HER2, human epidermal growth factor receptor 2; KPS, Karnofsky performance status; MLR, monocyte-to-lymphocyte ratio; NA, not available; SD, standard deviation; TNBC, triple-negative breast cancer.

* : p < 0.05.

**Table 5:** Changes in monocyte and lymphocyte counts changes according to BM LMR/primary LMR groups.

Variables	BM LMR/primary BRCA LMR=low	BM LMR/primary BRCA LMR=high	p
	(N=51)	(N=66)	
BM monocyte count / BRCA monocyte count (ratio)	3.1 ± 5.9	0.8 ± 0.5	0.003
BM lymphocyte count / BRCA lymphocyte count (ratio)	0.7 ± 0.4	1.5 ± 2.0	0.004

BM, brain metastasis; BRCA, primary breast cancer; MLR, monocyte-to-lymphocyte ratio.

## 4 Discussion

In this study of 117 patients with breast cancer who developed BM, we found that monocyte-related parameters, including LMR, and PMR, were significantly decreased at the time of BM compared with primary diagnosis. Importantly, a higher decrease in LMR was independently associated with poorer survival outcomes in multivariate analysis, together with TNBC subtype and the number of BMs. These findings suggest that peripheral blood monocyte dynamics may reflect clinically relevant changes during BM development.

In our multivariate analysis, traditional prognostic factors such as age and the presence of extracranial metastasis (ECM) did not reach statistical significance, whereas LMR transition emerged as a powerful independent predictor of overall survival. This finding suggests that in the advanced stage of BM, the dynamic shift in the systemic immune-inflammatory environment may be a more critical determinant of prognosis than baseline clinical characteristics. Unlike static parameters, the longitudinal LMR transition captures the host’s biological response to the metastatic process. Specifically, the significant surge in monocytes observed in the poor prognosis group (3.1-fold increase) underscores the dominant role of monocytic-driven immune suppression in determining survival. Within this high-risk patient population, the degree of immune-inflammatory collapse, as reflected by the LMR decline, appears to provide superior discriminatory power for predicting clinical outcomes compared to conventional prognostic markers.

Reportedly, the composition of peripheral blood cells, such as the NLR, LMR, and platelet-to-lymphocyte ratio, accurately reflects the prognosis of various solid cancers, including colorectal, lung, and breast cancer (12–17). Specifically, a high NLR was found to be closely associated with the occurrence of BM in BRCA and non-small cell lung cancer, correlating with a worse prognosis (18–20). This correlation has also been observed in our findings (**Supplementary Figure 2**), indicating that despite the retrospective nature of the study and the random timing of peripheral blood collection near the diagnoses of BRCA and BM, the analysis accurately reflects the prognosis of patients. In addition, based on our results, monocyte and monocyte-related ratios could be significantly associated with BM itself. Liu et al. reported a significant decrease in LMR in cases of BM when compared with BM in non-small cell lung cancer (21). Furthermore, the authors conducted a multivariate regression analysis and demonstrated that LMR is an independent risk factor contributing to BM (21). These findings are consistent with our observations. Additionally, our study suggests that the decrease in LMR at the time of BM diagnosis, compared with the values at primary BRCA diagnosis, is also clinically relevant, as it appears to substantially impact prognosis.

Growing clinical evidence suggests that CTC can induce CI during the BM process, potentially linked to the recruitment of monocytes and the regulation of the BBB (22–24). This evidence aligns with the findings reported by Kienast et al., suggesting that BM is a highly inefficient process, with only 1.0–2.4% of actual CTCs successfully establishing metastasis (4). In a study assessing 517 patients with lung cancer, Kato et al. observed that the incidence of CI was 2.9%, indicating a clinically similar pattern (25). The process of CI occurs due to the blockage of blood supply to the brain, leading to the migration of monocytes to the brain and their differentiation into macrophages (26, 27). During the initial stages, these primarily differentiate into M1 macrophages, secreting pro-inflammatory cytokines such as tumor necrosis factor-α, interleukin (IL)-1β, and IL-6, thereby promoting inflammation (28). Notably, these cytokines can compromise the integrity of the BBB. During the later stages of CI, monocytes differentiate into M2 macrophages, secreting anti-inflammatory cytokines such as IL-10 and transforming growth factor-β, mitigating inflammation and promoting brain recovery (29). Importantly, a weakened BBB integrity may facilitate the migration of CTCs to the brain, potentially leading to BM (30). Additionally, the involvement of monocyte-derived macrophages in the destruction of the BBB has been suggested (31). These macrophages may weaken the integrity of the BBB, facilitating the transit of tumor cells to the brain. Although this hypothesis provides important insights into the mechanisms of BM and CI, further research is necessary to fully understand the detailed mechanisms underlying these interactions.

Despite these insights, several limitations warrant consideration. First, as a single-center retrospective study with a relatively small cohort, our findings may have limited generalizability. The high exclusion rate (approximately 45%) was necessary to maintain a high-quality, longitudinal dataset by including only patients with available blood tests at both primary diagnosis and BM occurrence; however, this may introduce selection bias. Second, we lacked a comparator cohort of breast cancer patients without BM. Nevertheless, we employed a self-paired longitudinal design, allowing each patient to serve as their own control. This approach effectively minimizes inter-individual variability and focuses on the dynamic immune alterations specific to each patient’s clinical progression to BM. While matching a separate control group for age, subtype, and KPS is challenging and potentially bias-prone, future multicenter studies with external validation are needed to confirm the broader applicability of our internally derived LMR cutoff values. Finally, our proposed mechanism regarding monocyte-driven BBB permeability remains a hypothesis that requires further experimental validation. Nevertheless, despite these limitations, we believe that this research casts new light on the clinical importance of monocytes—previously under-recognized—and provides valuable insights into the mechanisms of BM.

## 5 Conclusion

This retrospective study suggests an association between decreased LMR at the time of BM diagnosis and poorer survival outcomes in patients with breast cancer. While our findings highlight a potential link between peripheral monocyte dynamics and BM, these results should be interpreted cautiously, as mechanistic inferences cannot be drawn without further experimental validation. Future studies with prospective design and biological analyses are warranted to clarify whether monocyte alterations play a causal role in BM development or simply reflect systemic disease progression.

## Data Availability

The original contributions presented in the study are included in the article/**Supplementary Material**. Further inquiries can be directed to the corresponding authors.
