# Supplementary material for: Peripheral blood monocytes could be associated with brain metastasis and affect patient prognosis in breast cancer: a retrospective study
Source: Front Oncol. 2026 May 21;16:1807911. doi: 10.3389/fonc.2026.1807911 (PMC13233264; doi:10.3389/fonc.2026.1807911)

**Supplementary Fig. S1.** STROBE-style flowchart of patient selection.

**Supplementary Fig. S2.** Neutrophil-to-lymphocyte ratio at diagnosis of primary breast cancer (BRCA) and diagnosis of brain metastasis (BM).


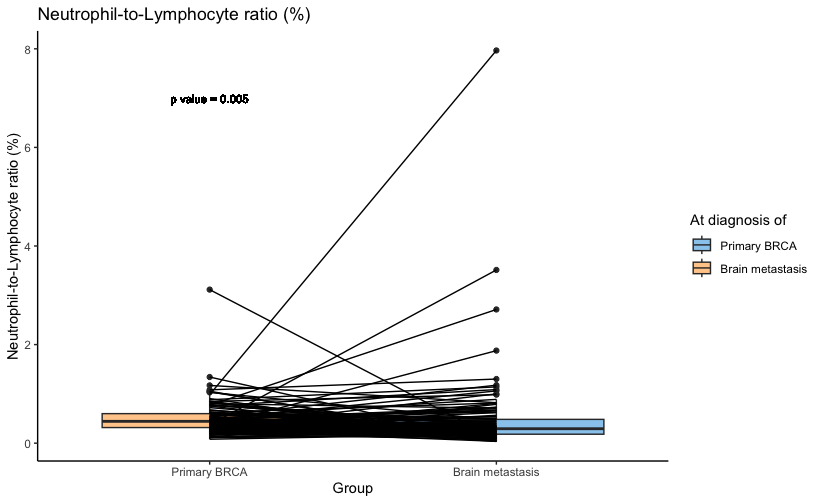

Supplement: Supplementary file 1 [file DataSheet1.docx]
